# A higher bacterial inward BCAA transport driven by *Faecalibacterium prausnitzii* is associated with lower serum levels of BCAA in early adolescents

**DOI:** 10.1186/s10020-021-00371-7

**Published:** 2021-09-15

**Authors:** Sofia Moran-Ramos, Luis Macias-Kauffer, Blanca E. López-Contreras, Hugo Villamil-Ramírez, Elvira Ocampo-Medina, Paola León-Mimila, Blanca E. del Rio-Navarro, Omar Granados-Portillo, Isabel Ibarra-Gonzalez, Marcela Vela-Amieva, Armando R. Tovar, Nimbe Torres, Francisco J. Gomez-Perez, Carlos Aguilar-Salinas, Samuel Canizales-Quinteros

**Affiliations:** 1 Consejo Nacional de Ciencia y Tecnología (CONACYT), Mexico City, Mexico; 2 Unidad de Genómica de Poblaciones Aplicada a la Salud, Facultad de Química, UNAM/Instituto Nacional de Medicina Genómica (INMEGEN), Periférico Sur No. 4809, Tlalpan, 14610 Mexico City, Mexico; 3 Hospital Infantil México Federico Gómez, Mexico City, Mexico; 4 Departamento de Fisiología de la Nutrición, Instituto Nacional de Ciencias Médicas y Nutrición Salvador Zubirán, Mexico City, Mexico; 5 Instituto de Investigaciones Biomédicas, UNAM - Instituto Nacional de Pediatría, Mexico City, Mexico; 6 Laboratorio de Errores Innatos del Metabolismo y Tamiz, Instituto Nacional de Pediatría, Mexico City, Mexico; 7 Departamento de Endocrinología y Metabolismo, Instituto Nacional de Ciencias Médicas Y Nutrición Salvador Zubirán, Mexico City, Mexico; 8 Unidad de Investigación en Enfermedades Metabólicas and Departamento de Endocrinología y Metabolismo, Instituto Nacional de Ciencias Médicas y Nutrición Salvador Zubirán, Mexico City, Mexico; 9 Tecnológico de Monterrey, Escuela de Medicina y Ciencias de la Salud, 64710 Monterrey, NL Mexico

**Keywords:** BCAA, Gut microbiome, Children, *Faecalibacterium prausnitzii*, Insulin resistance

## Abstract

**Background:**

Elevations of circulating branched-chain amino acids (BCAA) are observed in humans with obesity and metabolic comorbidities, such as insulin resistance. Although it has been described that microbial metabolism contributes to the circulating pool of these amino acids, studies are still scarce, particularly in pediatric populations. Thus, we aimed to explore whether in early adolescents, gut microbiome was associated to circulating BCAA and in this way to insulin resistance.

**Methods:**

Shotgun sequencing was performed in DNA from fecal samples of 23 early adolescents (10–12 years old) and amino acid targeted metabolomics analysis was performed by LC–MS/MS in serum samples. By using the HUMAnN2 algorithm we explored microbiome functional profiles to identify whether bacterial metabolism contributed to serum BCAA levels and insulin resistance markers.

**Results:**

We identified that abundance of genes encoding bacterial BCAA inward transporters were negatively correlated with circulating BCAA and HOMA-IR (*P* < *0.01*). Interestingly, *Faecalibacterium prausnitzii* contributed to approximately ~ *70%* of bacterial BCAA transporters gene count*.* Moreover, *Faecalibacterium prausnitzii* abundance was also negatively correlated with circulating BCAA (*P* = *0.001*) and with HOMA-IR (*P* = *0.018*), after adjusting for age, sex and body adiposity. Finally, the association between *Faecalibacterium* genus and BCAA levels was replicated over an extended data set (N = 124).

**Conclusions:**

We provide evidence that gut bacterial BCAA transport genes, mainly encoded by *Faecalibacterium prausnitzii*, are associated with lower circulating BCAA and lower insulin resistance. Based on the later, we propose that the relationship between *Faecalibacterium prausnitzii* and insulin resistance, could be through modulation of BCAA.

## Background

A high level of the branched-chain amino acids (BCAAs) leucine, isoleucine and valine has been viewed as a strong biomarker of obesity and insulin resistance in numerous studies, including adults and children (Fan and Pedersen 2021; Zhou et al. 2019) and is thought to be part of type 2 diabetes pathogenesis. Thus, accumulating evidence have focused on them as potential targets to improve insulin resistance and associated conditions (White and Newgard 2019).

The pathophysiology behind the elevated levels of BCAA is still controversial. On one hand, it has been show that BCAA catabolism in adipose tissue and liver is impaired in obesity and insulin resistance thus accounting for the increased circulating levels (Zhou et al. 2019). In addition, BCAA being essential amino acids are mainly obtained through the diet; however, its contribution to circulating levels is not clear. For instance, a couple of interventional studies have shown that a protein restricted or BCAA restricted diet can decrease circulating levels of these amino acids and improve insulin resistance markers (Fontana et al. 2016; Karusheva et al. 2019). In contrast, cross-sectional studies have shown that the amount or source of protein intake contributes only in a small percentage to circulating BCAA, suggesting that dietary intake of BCAA might not be the main variable affecting plasma levels of these amino acids (Jennings et al. 2016; Merz et al. 2018; Rousseau et al. 2019). Finally, a few studies in humans and animal models have shown that gut microbiota metabolism can contribute to BCAA synthesis, uptake and degradation and thus to its circulating levels (Liu et al. 2017, 2020; Pedersen et al. 2016; Ridaura et al. 2013). Particularly insulin resistant adults showed an increased potential to synthesize BCAA, which was largely driven by species such as *Prevotella copri* and *Bacteroides vulgatus* but a decreased potential for BCAA uptake and catabolism influenced by *Butirivibrio crossotus* and *Eubacterium siraeum* (Pedersen et al. 2016). However additional studies are needed to define the contribution of bacterial metabolism to the circulating pool of these metabolites, as well as the species contributing to these functions (White and Newgard 2019).

Gut microbiota structure and function has been shown to differ between adults and children (Hollister et al. 2015; Ringel-Kulka et al. 2013). Furthermore, we and others have shown that gut microbiota of early adolescents is still immature likely influencing its functionality (Moran-Ramos et al. 2020). However, fewer studies have explored the association of microbial metabolism to host circulating BCAA, in whom a different and immature gut microbiota, may yield different results (Radjabzadeh et al. 2020). For instance, in a cohort of Dutch school-age children a negative correlation between serum BCAA and the abundance of *Bacteroides vulgatus*, previously highlighted for its role in BCAA amino acid synthesis, was observed (Zhong et al. 2019), suggesting that the particular species contributing the bacterial metabolism of BCAA may vary among populations and/or life stages.

We have previously showed that circulating BCAA as part of an amino acid signature are associated with obesity, insulin resistance and higher serum triglycerides levels in Mexican children, without association with total dietary protein intake (Moran-Ramos et al. 2017). Thus, the aim of this work was to evaluate whether in early adolescents gut microbial metabolism contributes to circulating BCAA and its relation to insulin resistance.

## Materials and methods

### Study participants

This study was embedded in the “Obesity Research Study for Mexican Children” (ORSMEC), which is a population-based study in Mexico City including 6–12-year-old children recruited from a summer camp of children of employees of the Mexican Health Ministry (*Convivencia Infantil*, *Sindicato de la Secretaria de Salud*) and *Hospital Infantil de Mexico*. All children included reported no previous history of chronic medical illness, such as type 1 or type 2 diabetes, cancer or gastrointestinal diseases. Parents or guardians of each child signed the informed consent form and children assented to participate.

This study includes a subset of 23 children in the metagenomics analysis and 124 children for the replication analysis (extended dataset). All children were within the early adolescence stage (10–12 years old), provided a fecal sample and had no antibiotic use within the previous 3 months of sample collection. As previously described each participant was thoroughly assessed including demographic information and health-related phenotyped, via a self-administered questionnaire to the parents (including information about clinical history, current health status, and drug treatment) as well as a health assessment and extended clinical blood profiling included metabolomics. Microbiota of fecal samples from these children was previously characterized by 16S sequencing (Moran-Ramos et al. 2020).

For the validation analysis individuals were selected based on the original study design; age between 10 and 12 years old.

### Anthropometric and clinical parameters

Anthropometric measurements were determined as previously described (34) and included weight, height, waist and hip circumferences. Blood pressure (BP; mmHg) was measured using an automatic manometer (Microlife). Body fat mass percentage was obtained by bioelectrical impedance analysis (Quantum X Body Composition Analyzer, RJL Systems). Centers for Disease Control and Prevention 2000 growth charts were used as reference to determine body mass index (BMI) percentiles. Obesity status was defined as BMI percentile ≥ 95th, overweight between 85 and 95th percentile and normal-weight between the 5th and 85th percentile (Barlow and Expert 2007). National Heart, Lung, and Blood Institute reference data were used to determine BP percentiles based on height, age and gender (National High Blood Pressure Education Program Works Group on High Blood Pressure in C, Adolescents 2004). Metabolic syndrome was defined as established by De Ferranti (2004), with 3 or more of the following characteristics TG ≥ 100 mg/dL, HDL < 50 mg/dL, at least one of the BP percentiles > 90, glucose ≥ 110 mg/dL and waist circumference > 75th percentile.

### Blood sampling and biochemical analyses

Five mL blood samples were drawn after 8–12 h of fasting. For serum samples a 30-min period was allowed for clotting before serum separation and then stored at − 80 °C until further analysis. Serum levels of glucose, creatinine, uric acid, total cholesterol, HDL cholesterol, LDL cholesterol, TG, aspartate aminotransferase (AST), alanine aminotransferase (ALT), gamma glutamyl transpeptidase (GGT) and C-reactive protein (CRP) were measured with commercially available standardized methods (UNICEL DxC600, Beckman coulter). Insulin was determined using an Access 2 Immunoassay System (Beckman coulter). Insulin resistance was indirectly estimated with the homeostasis model assessment for insulin resistance (HOMA-IR) and calculated as ((fasting glucose (mg/dL) × fasting insulin (µU/mL))/405).

### Stool sampling and DNA extraction

Fecal samples were collected at home in a sterile cup, and refrigerated overnight prior to storage at − 70 °C until processing. DNA was extracted using the QIAamp® DNA Stool Mini Kit (Qiagen, Inc.), and stored at − 70 °C until further analysis. Concentrations of extracted DNA from each sample were determined by spectrophotometry (Nanodrop 2000c) measurement, and an estimate of sample purity was determined by measuring the A260/A280 absorbance ratio.

#### Microbiome taxonomic and functional potential profiling

The protocol for gut microbiota profiling by 16S sequencing was previously published (Moran-Ramos et al. 2020). Briefly the V4 hypervariable region was amplified using 515F and 806R primers, and the resulting libraries were sequenced using an Illumina MiSeq 2 × 250 platform. Sequences were analyzed using QIIME 1.9.0. Quality filters were used to remove sequences containing barcode mismatches, ambiguous bases, or low-quality reads (Phred quality score < 30). Operational taxonomic unit (OTU) read counts were calculated using the QIIME pipeline (75) (version 1.9.1; default parameters) with closed-reference OTU picking at 97% identity against the Greengenes database (version 13_08). Taxonomical classification was performed to generate phylum to genus level composition matrices.

For whole-genome shot-gun sequencing, 100 ng of DNA were used for the library preparation using the TruSeq DNA Nano protocol according to manufacturer’s instructions (Illumina Inc, San Diego, U.S.A.). Pair-end DNA sequencing (2 × 150) was performed on the Nextseq 500 Illumina platform. Raw Fastq files (average 21,076,621 reads per sample) were assessed for quality using Trimomatic, trimming adaptor reads and regions of quality below a Phred score of 33. The human reads (according to alignment to hg19, using BMTagger) or low-quality sequences were discarded.

Functional potential analysis was performed using HUMAnN2 (UniRef90 database), which computed the pathway profiles and gene family abundances, using ChocoPhlAn and UniRef90 databases (Franzosa et al. 2018). Differences in sequencing depth between samples were normalized in copies per million. To investigate the metabolic potential of the gut microbiome in relation to BCAA we used KEGG functional modules. Specifically, we manually searched for KEGG orthologous gene groups included in biosynthesis and inward transport pathways of theses amino acids from the KEGG database (Pedersen et al. 2016).

The taxonomic profiling and quantification of organisms’ relative abundances were quantified using MetaPhlAn2 (Segata et al. 2012) as this software has been shown to have higher precision than other read classifiers (Sczyrba et al. 2017).

### Metabolomic analysis

Serum amino acids were measured by a targeted metabolomics approach using electrospray tandem mass spectrometry (Quattro Micro API tandem MS), as previously described (Moran-Ramos et al. 2017). Leucine/isoleucine is reported as a single analysis because they are not resolved by our MS/MS method. Short-chain-fatty acids (SCFA) content in fecal samples were analyzed by gas chromatography (Agilent technologies-6850 series 11, Agilent, Santa Clara, CA, USA) with flame ionization detection (Agilent) and using Agilent J&W DB-225 ms column as previously described (Syeda et al. 2018).

### Statistical analysis

Statistical analysis was carried out using SPSS and R version 3.5.1. Continuous variables were presented as median and interquartile range (IQR) and compared using Kruskal–Wallis, while categorical variables were summarized as proportions and compared by Fisher’s exact test.

For functional profiles, we considered abundance of single microbial gene families and then normalized to counts per million (CPMs) to account for differences in sequencing depths, as provided by HUMAnN2. To estimate the linear correlations between gene abundance and metabolites or metabolic traits, we performed partial Spearman’s rank correlation analyses in R using *ppcor* package and controlling for age and sex, and when indicated for other confounding variables (i.e. body fat percentage, alpha diversity indices). P values were corrected for multiple testing using the Benjamini–Hochberg method as implemented in the *p.adjust* function in R (Benjamini and Hochberg 1995). Significance levels were defined at FDR < 5%.

For taxonomic abundances, we used the species-level relative abundances as estimated by MetaPhlAn v.2.0 (Segata et al. 2012). To determine taxa association with BCAA and metabolic traits, either partial Spearman’s correlations were calculated or the normalized abundance using the arcsine square-root transformation was used in linear regression models. In multiple linear regression models, taxa were used as explanatory variables. All models were controlled for age and sex, and when indicated for body fat percentage.

Finally, for replication analysis over the extended dataset, partial Spearman’s correlation coefficients were calculated to analyze bivariate relationships between 16S based taxonomy, serum BCAA and indicators of insulin resistance such as insulin, HOMA-IR and TG/HDL ratio (Giannini et al. 2011). Given that some subjects had missing values for body adiposity, correlations were controlled for age, sex, and when indicated for BMI percentile.

## Results

### Description of study population

**Fig. 1:**
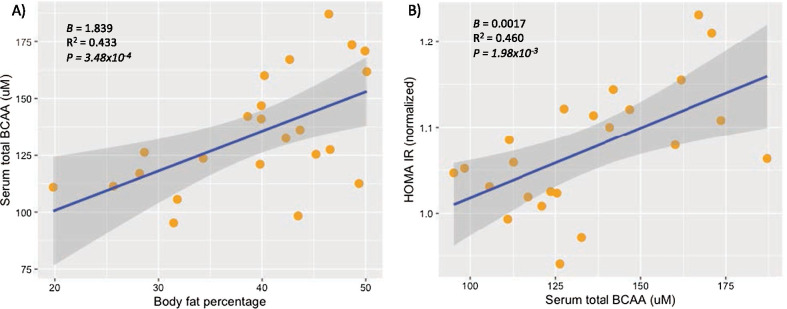
Association of total BCAA serum levels with the metabolic traits. **A** multiple linear regression models for the association between body fat percentage and serum levels of BCAA, adjusted for sex and age. **B** multiple linear regression models for the association between serum levels of BCAA and normalized values of HOMA IR, adjusted for sex and age

For our fecal metagenomic analysis we included 23 early adolescents (9 boys and 14 girls) with a median age of 11.7 (IQR 10.7–12.20 years). Seven were normal-weight, 8 overweight and 8 with obesity. In addition, out of the 16 children who were overweight/obese, half of them (n = 8) presented metabolic syndrome. Clinical and anthropometric characteristics of the included individuals are summarized in Table 1. Body fat percentage, biochemical parameters such as serum insulin, TG, HDL-C, and ALT levels as well as the HOMA-IR index were significantly different between normal-weight, overweight and obese children (Table 1). After adjusting for age and sex, higher serum levels of BCAA were significantly associated with greater body fat percentage (P = 3.48 × 10^–4^, Fig. 1A) and with higher HOMA-IR index (P = 1.98 × 10^–3^, Fig. 1B) and the latter association remained significant after further adjusting for body fat (P = 0.026).

**Table 1:** Clinical and biochemical characteristics of the study population

Trait	All subjects	Normal-weight	Overweight	Obese	P value
	(n = 23)	(n = 7)	(n = 8)	(n = 8)	
Sex (male %)	9 (39.1)	3(42.9)	3(37.5)	3 (37.5)	0.971
Age (years)	11.7 (10.7–12.20)	11.9 (10.8–12.3)	11.9 (11.0–12.4)	10.8 (10.5–11-8)	0.122
Clinical					
Height percentile	67.1 (58.4–74.3)	65.6 (38.2–76.4)	73.8 (65.0–82.3)	60.1 (49.6–69.7)	0.078
BMI percentile	92.0 (77.7–95.4)	59.0 (10.1–77.0)	91.6 (89.4–93.6)	96.3 (95.3–97.1)	**5.6 × 10**^**–5**^
Body fat (% of BW)	40.2 (31.8–46.4)	28.6 (25.65–31.82)	40.1 (39.8–44.7)	47.6 (42.9–49.8)	**2.1 × 10**^**–4**^
Systolic BP percentile	67.3 (30.5–80)	25.8 (22.2–35.7)	71.2 (39.4–81.3)	67.6 (63.0–85.9)	0.066
Diastolic BP percentile	81.2 (63.2–89.9)	63.2 (53.5–82.6)	85.0 (74.3–92.5)	80.5 (68.1–91.5)	0.272
Biochemical					
Glucose (mg/dL)	89 (86–93)	89.0 (86.0–89.0)	89.5 (86.5–94.5)	89.0 (85.5–93.3)	0.866
Insulin (µU/mL)	8.20 (5.80–13.5)	6.10 (4.40–7.10)	10.4 (6.2–14.4)	13.4 (8.08–27.2)	**0.029**
HOMA IR	1.85 (1.26–3.12)	1.28 (0.93–1.58)	2.37 (1.36–3.14)	2.86 (1.79–6.09)	**0.037**
CRP (mg/dL)	0.12 (0.03–0.33)	0.05 (0.01–0.29)	0.11 (0.03–0.28)	0.43 (0.07–0.96)	0.105
Creatinine (mg/dL)	0.51 (0.49–0.56)	0.52 (0.45–0.56)	0.50 (0.48–0.56)	0.52 (0.50–0.58)	0.682
Uric acid (mg/dL)	5.20 (4.50–6.20)	4.40 (3.70–5.40)	5.25 (4.83–6.03)	5.95 (5.20–6.55)	0.062
Lipids					
Triglycerides (mg/dL)	81.0 (62.0–112)	63.0 (41.0–66.0)	91.0 (69.0–138.3)	95.5 (66–133.75)	**0.040**
Total cholesterol (mg/dL)	162 (142–177)	159 (135–177)	175 (144 -193)	161 (134–179)	0.769
HDL-C (mg/dL)	44.0 (35–53)	53.0 (48.0–58.0)	43.0 (34.3–49.0)	38.0 (31.3–43.5)	**0.006**
LDL-C (mg/dL)	97.5 (78.8–116)	94.0 (70.0–112)	101.5 (80.0 -125)	102 (78.0–126)	0.810
TG to HDL ratio	1.88 (1.31–2.80)	1.18 (0.64–1.43)	2.11 (1.45–4.04)	2.35 (1.81–4.34)	**0.009**
Liver enzymes					
AST (UI/L)	27.0 (22.0–29.0)	27.0 (22.0–29.0)	22.0 (19.3–27.0)	30.0 (22.8–37.3)	0.056
ALT (UI/L)	19.0 (15.0–35.0)	19.0 (15.0–22.0)	15.0 (14.0–16.8)	35.0 (26.0–44.5)	**0.002**
GGT (UI/L)	14.0 (12.0–15.0)	12.0 (11.0–15.0)	13.5 (12.0–14.75)	14.5 (13.3–18.0)	0.134
Serum amino acids					
Leucine/Isoleucine (μM)	59.9 (50.8–67.0)	50.8 (47.7–55.5)	62.1 (53.4–65.5)	67.4 (61.7–75.1)	**0.009**
Valine (μM)	69.7 (64.9–88.8)	61.8 (57.9–66.9)	72.5 (65.8–81.5)	94.9 (70.0–98.3)	**0.005**
BCAA (μM)	127.5 (112.6–160.1)	111.4 (105.6–123.6)	134.3 (122.2–145.7)	164.5 (133.5–172.9)	**0.004**

Data are shown as medians (interquartile range) or n (%). P-value was obtained using Kruskal–Wallis test or Fisher’s exact test for categorical variables. Significant P-values are shown in bold

*BMI* body mass index, *HDL-C* high-density lipoprotein cholesterol, *LDL-C* low-density lipoprotein cholesterol

#### Contribution of gut bacterial metabolism to serum BCAA

**Fig. 2:**
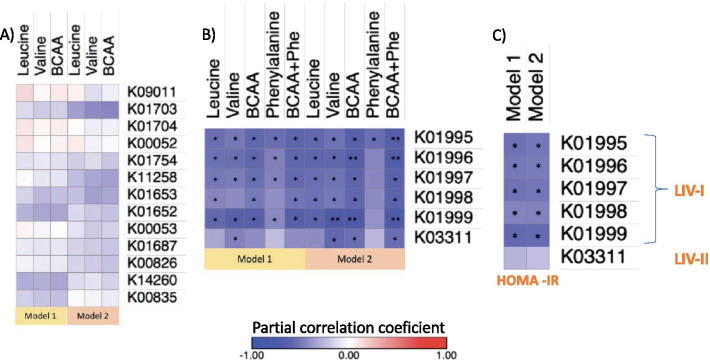
Association of bacterial gene abundance with BCAA serum levels and HOMA-IR. **A** heat map of the partial Spearman’s rank correlation coefficient between genes involved in BCAA biosynthesis and serum levels of BCAA. **B** heat map of the partial Spearman’s rank correlation coefficient between genes involved in BCAA transport and serum levels of BCAA. **C** heat map of the partial Spearman’s rank correlation coefficient between genes involved in BCAA transport and HOMA-IR. P-values were calculated using partial correlations adjusted for Model 1: age and sex. Model 2: Model 1 + body fat percentage. False discovery rate (FDR) adjusted *P < 0.05, **P < 0.01

Gut microbiota is involved in the synthesis and uptake of BCAA, thus, we investigated the metabolic potential of the gut microbiome in relation to these metabolites using KEGG functional modules. In the gut metagenomes analyzed by the HUMANn2 algorithm, we identified 14 out of 17 gene families encoding the pathway for the biosynthesis of leucine, valine and isoleucine (Additional file 1: Table 1). To study the association between the identified biosynthesis genes and serum levels of BCAA, we performed partial spearman correlations. However, none of the correlations with individual or total BCAA were significant, after adjusting for age and sex or after further adjustment for body adiposity (Fig. 2A).

We then searched for the bacterial genes involved in the inward bacterial transport of BCAA. We identified six gene families encoding two BCAA transporters: the high affinity LIV-I/LS and the low affinity LIV-II (*BrnQ*) transporter (Additional file 1: Table 2). To determine whether the gene families were associated with circulating BCAA we calculated Spearman correlation coefficients controlling for age and sex. As shown in Fig. 2B, after correcting for multiple testing most of the transport genes from LIV-I system were negatively correlated with individual and total BCAA as well as with phenylalanine that is also a substrate transported by this system. After further controlling for body fat percentage, these correlations remained significant for individual and total BCAA (FDR P < 0.05) and trended for phenylalanine (FDR P < 0.1). Abundance of the LIV-II transporter gene was also negatively correlated with serum levels of valine and remained significant after adjusting for body fat percentage (P < 0.05).

Given the relation between BCAA and insulin resistance we then sought whether transporter genes were also associated with HOMA-IR (an insulin resistance marker). As observed in Fig. 2C, only the abundance of genes encoding the high affinity LIV-I/LS system were negatively correlated with HOMA-IR and the correlations were still significant after adjusting for body fat percentage. To gain insight on whether the association between genes encoding de LIV-I/LS system and HOMA-IR was related to serum levels of BCAA, we adjusted the correlation by the effect of total BCAA serum levels. Accordingly, all correlations lost significance (P > 0.5, Additional file 1: Table 3).

**Fig. 3:**
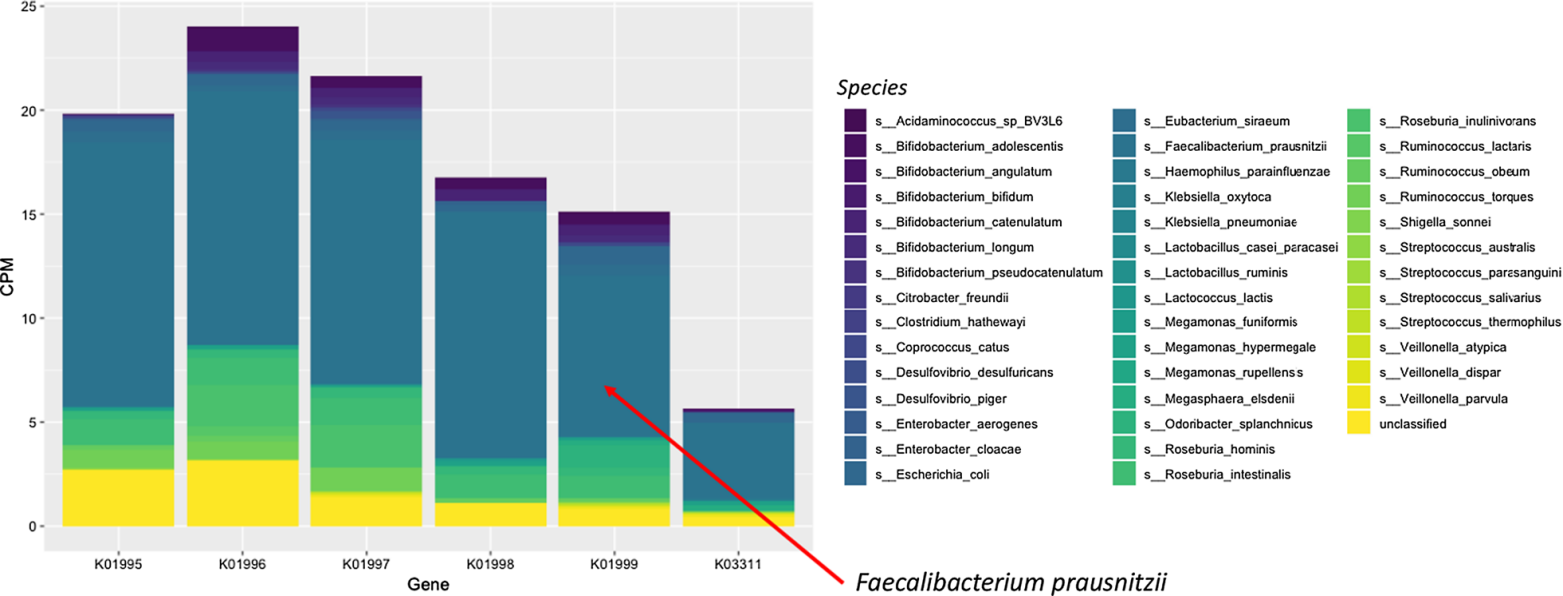
Mean abundance of bacterial transport gene families stratified by species. The plot includes the 42 species encoding at least one of the gene families and the counts explained by unclassified taxa. *CPM* counts per million

One of the features of HUMAN2 is the ability to compute gene family abundance stratified by bacterial species to assess per-organism contribution (Franzosa et al. 2018). Thus, we then assessed which bacterial species were encoding the transport genes. The analysis showed that these genes were present in 42 out of 189 identified metagenomic species belonging mainly to the Firmicutes phylum as well as to *Enterobacteriaceae* and *Bifidobacteriaceae* families (Fig. 3). Of the 42 bacterial species, only ten showed counts for the five necessary genes to encode the whole LIV-I/LS transport system and to functionally mediate transport into the bacterial cytoplasm (Adams et al. 1990). Interestingly, 90% of the gene counts belonged only to two species; *Faecalibacterium prausnitzii* which accounted for 70–80% and *Roseburia hominis* that contributed to 8–10% of the counts*.*

#### Correlation of metagenomic species with BCAA circulating levels and insulin resistance

**Fig. 4:**
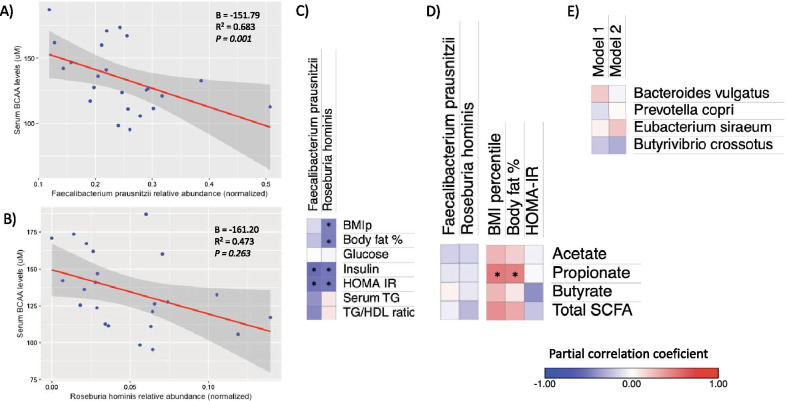
Associations of metagenomic species with serum BCAA, metabolic traits and fecal SCFA. **A**, **B** multiple linear regression models for the association between the arcsin-sqrt normalized abundance of *Faecalibacterium* and *Roseburia* species and serum BCAA levels adjusting for age, sex and body fat percentage. **B** heat map of the partial Spearman’s rank correlation coefficient between species relative abundance and metabolic traits. **C** Heat map of the partial Spearman’s rank correlation coefficient of fecal SCFA concentrations with species abundance and metabolic traits. **A**, **B** regression models were adjusted for age, sex and body fat percentage, gray shading represents 95% CI. **C**, **D** partial correlations for anthropometric traits were adjusted for age and sex, and for biochemical variables were further adjusted for body adiposity. **E** heat map of the partial Spearman’s rank correlation coefficient between selected species and total serum BCAA levels. P-values were calculated using partial correlations adjusted for Model 1: age and sex. Model 2: Model 1 + body fat percentage. *P < 0.05. *BCAA* branched-chain amino acids, *SCFA* short-chain fatty acids

Given that only two species encoded for the majority of uptake gene counts (~ 90%), we sought to determine if the abundance of these species were associated with BCAA circulating levels, body fat and insulin resistance. *Faecalibacterium prausnitzii* was present in all individuals and showed median abundance of 5.8%, while *Roseburia hominis* was present in 22/23 individuals but show a lower abundance (median = 0.13%). In a multivariate linear regression analysis, greater relative abundance of *Faecalibacterium prausnitzii* was associated with lower serum BCAA levels (P = 1.4 × 10^–3^, Fig. 4A). Furthermore, *Faecalibacterium prausnitzii* abundance was also negatively correlated with serum insulin levels and HOMA-IR (P < 0.05), and as a trend with the TG/HDL ratio (P = 0.053; Fig. 4C). The former correlation was lost when adjusting for serum total BCAA (P > 0.3). On the other hand, greater abundance of *Roseburia hominis* was associated with a lower body fat percentage, insulin levels and HOMA-IR (P < 0.05, Fig. 4C). However, in the regression model the association with BCAA levels lost significance when adjusting for body adiposity (P = 0.26, Fig. 4B).

Among previously reported bacterial species for its contribution to circulating BCAA are *Bacteroides vulgatus*, *Prevotella copri*, *Eubacterium siraeum* and *Butyrivibrio crossotus* (Pedersen et al. 2016). Although in our sample, abundance of these species was detected, none of them showed a significant correlation with total BCAA levels (Fig. 4E).

#### Role of short-chain fatty acids in the association between butyrate producer species and circulating BCAA

*Faecalibacterium prausnitzii* as well as *Roseburia hominis* are considered predominant butyrate producers (Louis and Flint 2017). Indeed, one of the mechanisms by which *Faecalibacterium prausnitzii* has been related to a better gastrointestinal and metabolic health is through its butyrate production capacity (Leylabadlo et al. 2020). Thus, to assess whether butyrate levels could be also contributing to the observed associations with insulin resistance markers, we measured fecal short-chain fatty acids (SCFA). According to previous studies the fecal concentration of SCFA was acetate > propionate > butyrate (Additional file 1: Table 4). Total fecal SCFA were higher in overweight/obese vs normal-weight children although the significance was only as a trend (P = 0.091, Additional file 1: Table 4). Interestingly even though fecal butyrate concentration showed a negative correlation with HOMA-IR (P = 0.096), neither *Faecalibacterium prausnitzii* nor *Roseburia hominis* abundance showed an association with fecal butyrate concentration (P > 0.7, Fig. 4D).

#### Replication of *Faecalibacterium* associations with BCAA and insulin resistance markers over an extended dataset with 16S rRNA data

To validate the associations of the taxa harboring BCAA transporters genes with circulating levels of BCAA and insulin resistance markers, we pursued replication over an extended dataset of 124 children with 16S rRNA sequencing data, including the 23 individuals with metagenomic shotgun sequencing who also had 16S rRNA data.

**Fig. 5:**
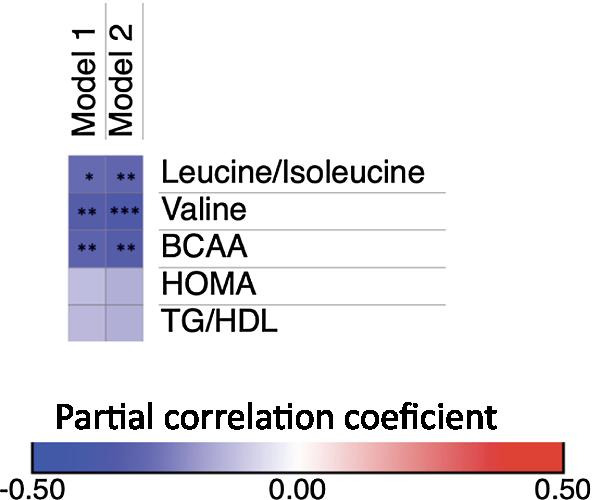
Association of 16S *Faecalibacterium* genus abundance with BCAA serum levels and insulin resistance markers in the extended dataset (N = 124). P-values were calculated using partial spearman correlations adjusted for Model 1: age and sex. Model 2: Model 1 + BMI percentile. *P < 0.01, **P < 0.01, ***P < 0.001

*Faecalibacterium prausnitzii* is the only identified specie within the *Faecalibacterium* genus (Filippis et al. 2020), while *Roseburia hominis* is one of the five identified species within the genus *Roseburia* (Tamanai-Shacoori et al. 2017). Thus, considering that in the original dataset (n = 23), metagenomic abundance of *Faecalibacterium prausnitzii* showed a fairly good correlation with the abundance of *Faecalibacterium* genus obtained through 16S sequencing (rho = 0.790, P = 7.20 × 10^–6^), we sought whether the associations between *Faecalibacterium* genus, circulating BCAA and the insulin resistance markers, were still valid. In the extended dataset, fecal samples showed a similar *Faecalibacterium* median abundance (4.16%). Consistently with the metagenomics results, *Faecalibacterium* abundance showed a negative correlation with individual and total serum BCAA levels (Leucine/Isoleucine rho = − 0.281, Valine rho = − 0.333, total BCAA rho = − 0.310, P < 0.01, Fig. 5), after adjusting for age and sex. Moreover, these correlations remained significant after adjusting for BMI percentile (P < 0.01, Fig. 5). *Faecalibacterium* abundance also showed a negative but not significant correlation with HOMA-IR after adjusting for BMI percentile (rho = − 0.146, Fig. 5). Noteworthy, the negative correlation of *Faecalibacterium* genus with individual and total BCAA remained significant after excluding the 23 children with shotgun metagenomic data (Total BCAA rho = − 0.310, *P* = 0.001), while the correlation with HOMA although negative was not significant (rho = − 0.117, *P* = 0.24).

## Discussion

A relationship between the gut microbiome and circulating BCAA is already established (Liu et al. 2017, 2020; Pedersen et al. 2016; Ridaura et al. 2013), however details about specific taxa contributing to this association in different populations are lacking, particularly in pediatric groups. By using shotgun sequencing we aimed to evaluate the contribution of bacterial metabolism, to circulating BCAA in a sample of Mexican early adolescents and whether this was also related to insulin resistance.

Interestingly we showed that it was the higher gene abundance of inward bacterial transporters of BCAA that was strongly related with lower circulating levels of these amino acids. Although previous studies have also showed that biosynthesis pathways contribute to higher levels of BCAA (Pedersen et al. 2016; Dhakan et al. 2019), we did not observe any association between circulating levels of these amino acids and bacterial genes involved in BCAA biosynthesis pathways or with species previously reported to be involved in these processes. This could be somewhat expected as the gene repertoire, as well as the distribution of different strain patterns within species, respond to extrinsic factors such as geography, age, diet or even host genetics (Filippis et al. 2019; Truong et al. 2017). In spite of this, our results are consistent with a previous report showing that in a Southern Indian population, where habitual diet is composed of rice as well as meat and fish, higher gene abundance of bacterial BCAA inward transporters correlated well with lower serum BCAA concentrations, and in addition with higher levels in feces (Dhakan et al. 2019). Interestingly, in the same a study, in a Northern Indian population, with a diet enriched in carbohydrates and trans-fat foods, higher bacterial abundance of BCAA biosynthesis genes correlated with greater serum levels of these amino acids and lower fecal levels. Although we did not measure fecal BCAA levels, the former and our results suggest that higher uptake of BCAA by gut bacteria could decrease their bioavailability and thus their circulating levels. Furthermore, it underscores the relevance for future studies to assess whether the microbiome functional potential is also related to certain dietary habits.

Interestingly we further observed that gene abundance of the high affinity bacterial BCAA transporter LIV-I, also showed a strong correlation with HOMA-IR. Although at a gene cluster level our results are in agreement with other reports, the specific species that contribute to bacterial transporters gene abundance are not always the same. In our sample, the main species harboring these genes were *Faecalibacterium prausnitzii* and *Roseburia hominis*. *Faecalibacterium* is among the taxa associated with the transport gene clusters in the Indian adult population study, which suggests that our finding is not exclusive of pediatric populations. However, the species identified in other reports (Pedersen et al. 2016), such as *Eubacterium siraeum* or *Butirivibrio crossotus* showed a low prevalence or abundance in the children’s fecal bacterial community and when present they did not seem to contribute to bacterial gene count.

*Faecalibacterium prausnitzii* is considered a beneficial microbe that although not always consistently among studies, has been associated with lower insulin resistance and it is decreased in subjects with T2D (Gurung et al. 2020). In our study, greater abundance *of Faecalibacterium prausnitzii* was associated with both; lower serum BCAA and lower HOMA-IR, and the latter association was lost when adjusting for total BCAA levels. The former finding was replicated over an extended dataset (n = 124), providing novel evidence for a possible role of *Faecalibacterium prausnitzii* in the host BCAA metabolism.

BCAA have linked to insulin sensitivity through an mTOR mediated pathway and the production of 3-hydroxyisobutirate, a catabolic intermediate of BCAA (Jang et al. 2016; Newgard et al. 2009). However recent studies have shown that the balance between BCAA and other essential amino acids such as tryptophan and threonine also play a role in the metabolic derangements produced by higher BCAA (Solon-Biet et al. 2019). Indeed, in a previous report we showed that a metabolomic signature composed of BCAA but also aromatic amino acids as well as alanine and proline was associated with insulin resistance and future development of hypertriglyceridemia (Moran-Ramos et al. 2017). Intriguingly and despite that for the extended dataset, individuals were selected based on the original study design; in terms of age, and adiposity proportions, the association between *Faecalibacterium* abundance and HOMA-IR did not reach statistical significance. When comparing individuals from the metagenomic study vs those in the extended data set, we observed some metabolic differences. Thus, these phenotype differences could somehow confound the result.

Interestingly a recent study in a murine model showed that dietary BCAA restriction from an early age, besides promoting metabolic health trough out life stages; it also prevents age-associated frailty and increases lifespan, particularly in males (Richardson et al. 2021). Thus, it would be interesting to investigate whether the contribution of bacterial metabolism to host BCAA could have effects beyond metabolic health.

The proposed classical mechanisms for the association between *Faecalibacterium prausnitzii* and metabolic health have been related to its anti-inflammatory potential (Leylabadlo et al. 2020). Butyrate is one of the main metabolic end-products of *Faecalibacterium prausnitzii* fermentation. This metabolite is known to possess anti-inflammatory activity and improve gut barrier integrity, which might modulate insulin resistance derived from chronic low-grade inflammation (Chambers et al. 2018). However, in our samples the abundance of *Faecalibacterium prausnitzii* was not associated with fecal butyrate concentrations. The latter further emphasizes that in our population, the association between *Faecalibacterium prausnitzii* and HOMA-IR could be mediated by its contribution to BCAA metabolism. The products of bacterial fermentation of BCAA are branched short-chain fatty acids (BSCFA). Although little is known regarding the role of gut-derived BSCFAs in the regulation of metabolism, there is some evidence that through their effects on adipocytes they could contribute to an improved insulin sensitivity (Heimann et al. 2016). Thus, it would be of interest to assess whether BSCFAs, are associated with a greater abundance of *Faecalibacterium* as well as to lower levels of insulin resistance markers.

Although at a functional level *Faecalibacterium* genus has not been previously recognized for its role in BCAA metabolism, an in vitro study showed that a particular strain of *Faecalibacterium* was able to consume BCAA (Heinken et al. 2014). A few other studies assessing gut microbiota by 16S sequencing have observed indirect associations. For instance, in a study including Indian and Chinese adults, *Faecalibacterium* abundance show a negative correlation with BCAA urine levels (Jain et al. 2019). Likewise, in an animal model with a dietary intervention with inulin, an increase in *Faecalibacterium* abundance was observed, and in parallel a reduction in serum BCAA as well as an increase in fecal isobutyrate levels, a main product of BCAA fermentation (Wu et al. 2020). In vitro experiments also show ability of certain *Faecalibacterium prausnitzii* strains to metabolize fibers, directly or indirectly, through metabolic cross-feeding. We did not performed associations with dietary habits, however the former findings further acknowledge the need of future studies to understand the interaction between the dietary components and *Faecalibacterium* growth or metabolism. Whether the transport of BCAA by *Faecalibacterium* is an adaptative response to high BCAA diets or if certain type of diets promote its growth is still warranted. Furthermore, in the light of the beneficial effects of this specie it might be relevant to evaluate dietary interventions as a potential strategy to increase *Faecalibacterium* abundance and thus modulate serum BCAA levels.

Finally, it was recently reported that although *Faecalibacterium prausnitzii* is the only identified species within the genus, there are 22 clades with different functional potential that are differentially distributed not only among populations but also throughout life stages (Filippis et al. 2020). Thus, the reasons for the scarce reported associations between this bacterial species and BCAA levels could rely on the observed high level of functional diversity and specialization of *Faecalibacterium prausnitzii* strains (Fitzgerald et al. 2018). Although in our population *Faecalibacterium prausnitzii* seems to be particularly relevant for BCAA metabolism, further studies are needed to identify which particular clades or strains are linked to this function.

## Limitations of the study

We acknowledge several limitations in our study. First it was a cross-sectional investigation with a relatively small sample size, which could have influenced the lack of association between bacterial BCAA biosynthesis pathways and serum levels of these amino acids and does not establish causality. Second there are other factors that influence serum BCAA levels, such as genetic background and long-term dietary intake, that were not assessed in our study. Even though in a previous study we showed that the serum amino acid signature was not related to dietary protein intake (Moran-Ramos et al. 2017), it will be important for future studies to assess whether BCAA or animal protein intake rather that protein intake itself could contribute to serum BCAA levels as well as to the relative abundance of *Faecalibacterium*. Third, the results may not be applicable to individuals of other geographic origins or life stages. Even though the median abundance of the children included in the study is similar to other school-aged children. A recent study showed that *Faecalibacterium* abundance and diversity are different among children and adults, and between westernized and not-westernized populations (Filippis et al. 2020). Thus, whether the observed associations are also valid in adults or in children from other geographic regions warrants further studies. Fourth while shotgun sequencing allowed us to infer the functional capability of the gut microbial community, it does not provide information of the actual active genes. A metatranscriptomics or metaproteomics approach will help in bridging this gap, however in vitro studies as well as an animal model would be required to establish causality between *Faecalibacterium prausnitzii* abundance and host BCAA levels in different nutritional settings.

Notwithstanding the above limitations, the validated association between *Faecalibacterium* abundance and serum BCAA levels, over an extended data set, strengthen the metagenomic results and add to the evidence for a novel role of *Faecalibacterium* in BCAA metabolism and possibly for the pathogenesis of insulin resistance.

## Conclusions

In conclusion, we described an inverse association between bacterial BCAA inward transport genes and serum levels of these amino acids. We suggest that through this pathway the gut microbiome could contribute to lower BCAA levels in circulation. In addition, because there is no direct evidence of the role of *Faecalibacterium prausnitzii* in human BCAA metabolism, here, we showed for the first time that the relationship between *Faecalibacterium prausnitzii* and insulin resistance, could be through modulation of BCAA.

## Supplementary Information

**Additional file 1: Table S1.** Identified KEGG Orthologs involved in bacterial BCAA biosynthesis. **Table S2.** Identified KEGG Orthologs involved in bacterial BCAA transport. **Table S3.** Partial Spearman Correlations between inward transport gene abundance and HOMA-IR levels. **Table S4.** Concentration of fecal short-chain fatty acids. **Table S5.** Characteristics of the Mexican early adolescents included in the extended dataset.

## Data Availability

The datasets analyzed during the current study are not publicly available because in the consent form participants were not asked for their data to be shared publicly, however they are available from the corresponding author on reasonable request.
